# Supplementary figures and images for: SRSF2 is required for mRNA splicing during spermatogenesis
Source: BMC Biol. 2023 Oct 23;21:231. doi: 10.1186/s12915-023-01736-6 (PMC10591377; doi:10.1186/s12915-023-01736-6)

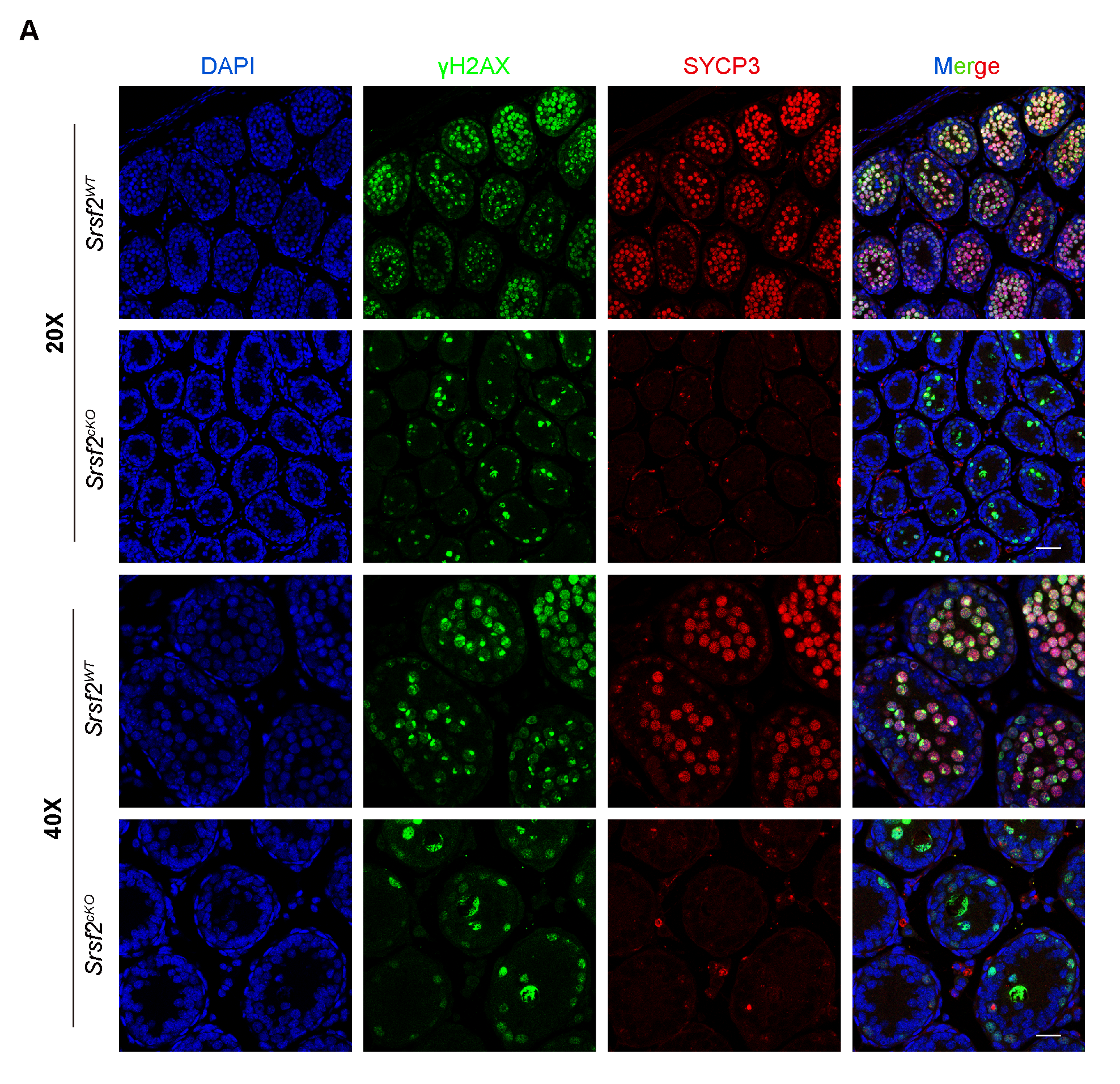

Supplement: Supplementary file 1 — Additional file 1: Fig. S1. Spermatogenesis fails to progress into meiosis in Srsf2 deficient germ cells at P12 γH2AX (green) and SYCP3 (red) immunofluorescence analysis of the Srsf2WT and Srsf2cKO male mice at P12. Scale bar: (top) 50 μm; (bottom) 20 μm. [file 12915_2023_1736_MOESM1_ESM.tif]

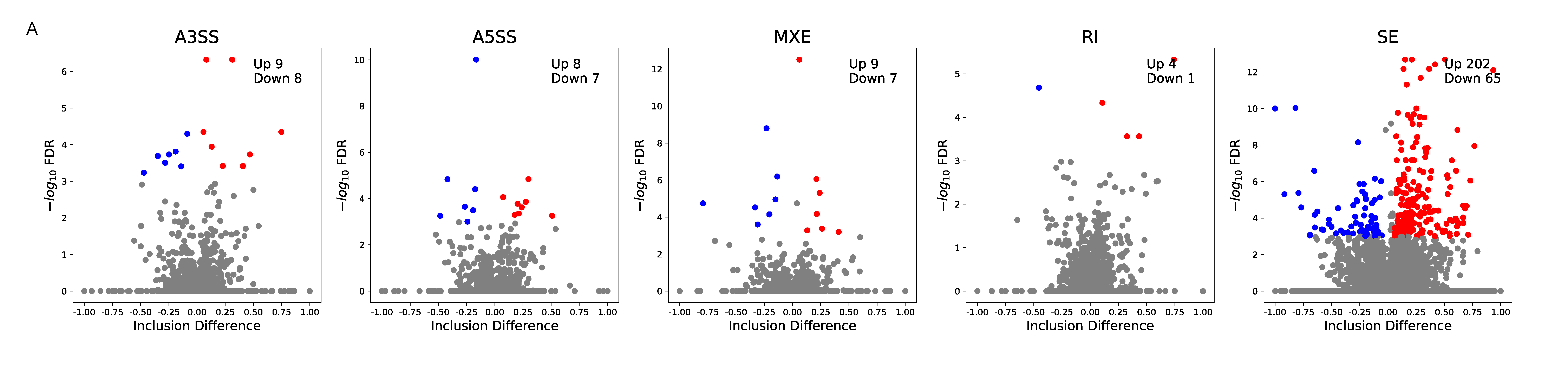

Supplement: Supplementary file 2 — Additional file 2: Fig. S2. SRSF2 regulates mRNA alternative splicing in testes Five AS events significantly affected by deletion of SRSF2 in the testes at P10. The different types of alternatively spliced events were shown. [file 12915_2023_1736_MOESM2_ESM.tif]

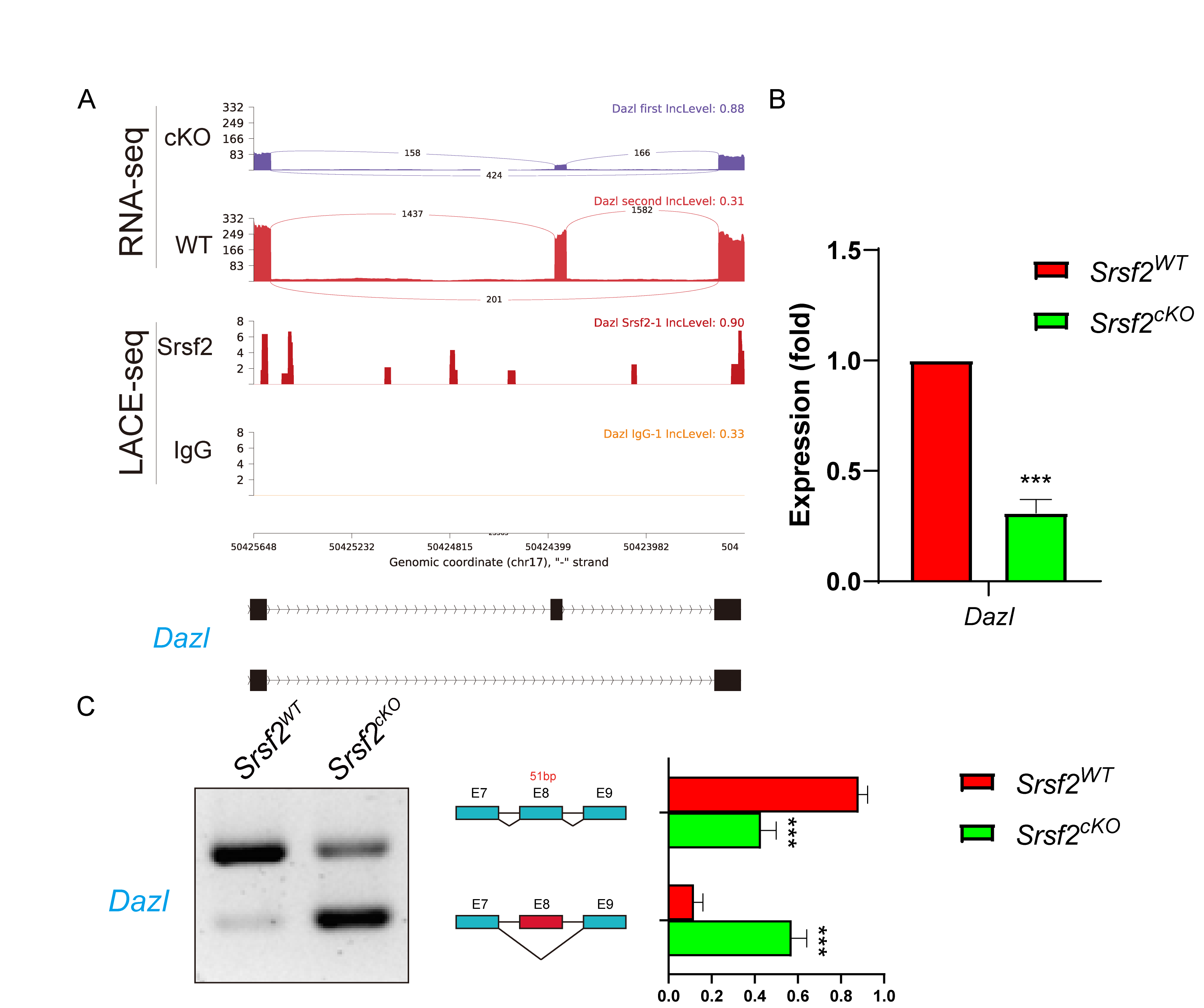

Supplement: Supplementary file 3 — Additional file 3: Fig. S3. SRSF2 indirectly regulates splicing and expression of Dazl. (A) A magnified view showing RNA-seq signals of the Dazl gene. (B) Quantitative RT-PCR validation of the expression of Dazl. (C) Semiquantitative RT-PCR analysis of AS patterns of the changed spliced genes in Srsf2WT and Srsf2cKO testes at P10 (n = 4 per group). PCR primers are listed in Additional file 5: Table S1. The scheme and cumulative data on percentage of the indicated fragment are shown accordingly. [file 12915_2023_1736_MOESM3_ESM.tif]

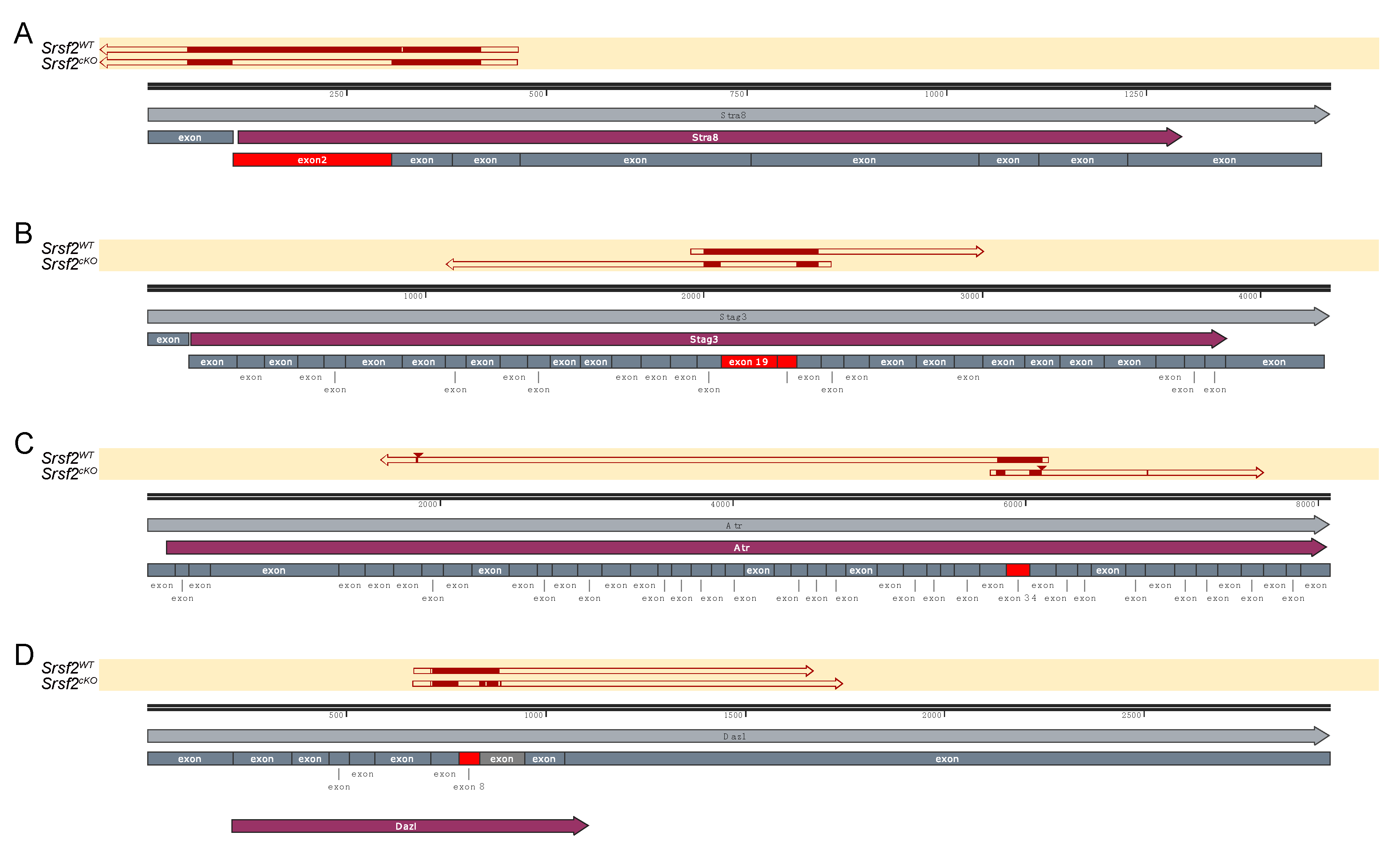

Supplement: Supplementary file 4 — Additional file 4: Fig. S4. The effect of SRSF2 deletion-introduced exon skipping. (A) The effect of SRSF2 deletion-introduced exon 2 skipping on Stra8 mRNA. The sequence of targeted exon was showed between Srsf2WT and Srsf2cKOgroups. (B) The effect of SRSF2 deletion-introduced exon 19 and exon 20 skipping on Stag3 mRNA. The sequence of targeted exon was showed between Srsf2WT and Srsf2cKOgroups. (C) The effect of SRSF2 deletion-introduced exon 34 skipping on Atr mRNA. The sequence of targeted exon was showed between Srsf2WT and Srsf2cKO groups. (D) The effect of SRSF2 deletion-introduced exon 8 skipping on Dazl mRNA. The sequence of targeted exon was showed between Srsf2WT and Srsf2cKO groups. [file 12915_2023_1736_MOESM4_ESM.tif]

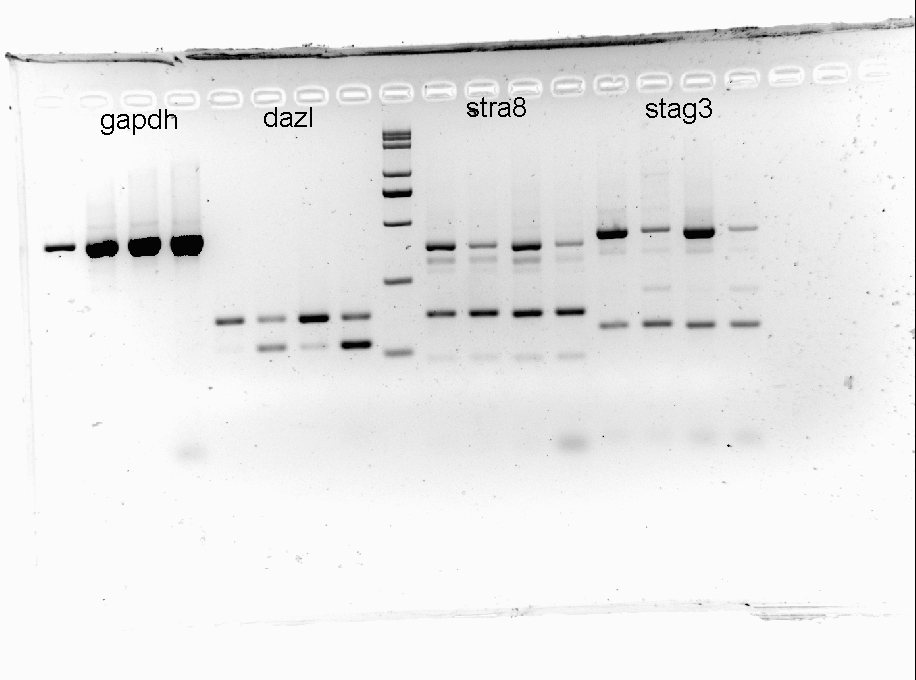

Supplement: Supplementary file 8 — Additional file 8. Images of the original, uncropped gels/blots. [file 12915_2023_1736_MOESM8_ESM.zip › Additional file 8.Images of the original, uncropped gelsblots/Additional file 3 Fig. S3-Dazl.jpg]

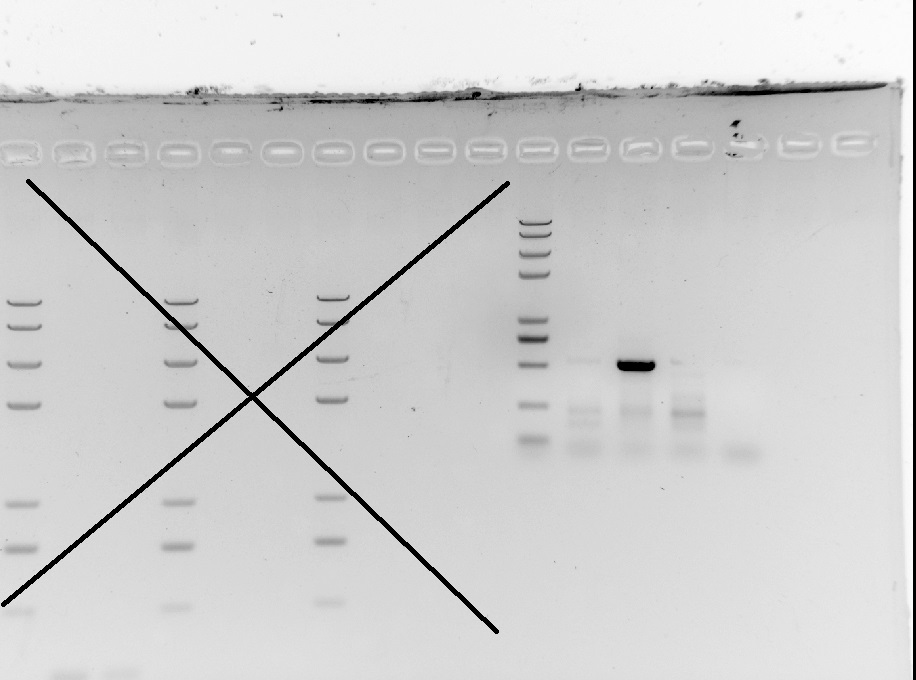

Supplement: Supplementary file 8 — Additional file 8. Images of the original, uncropped gels/blots. [file 12915_2023_1736_MOESM8_ESM.zip › Additional file 8.Images of the original, uncropped gelsblots/Fig 1C-delta.jpg]

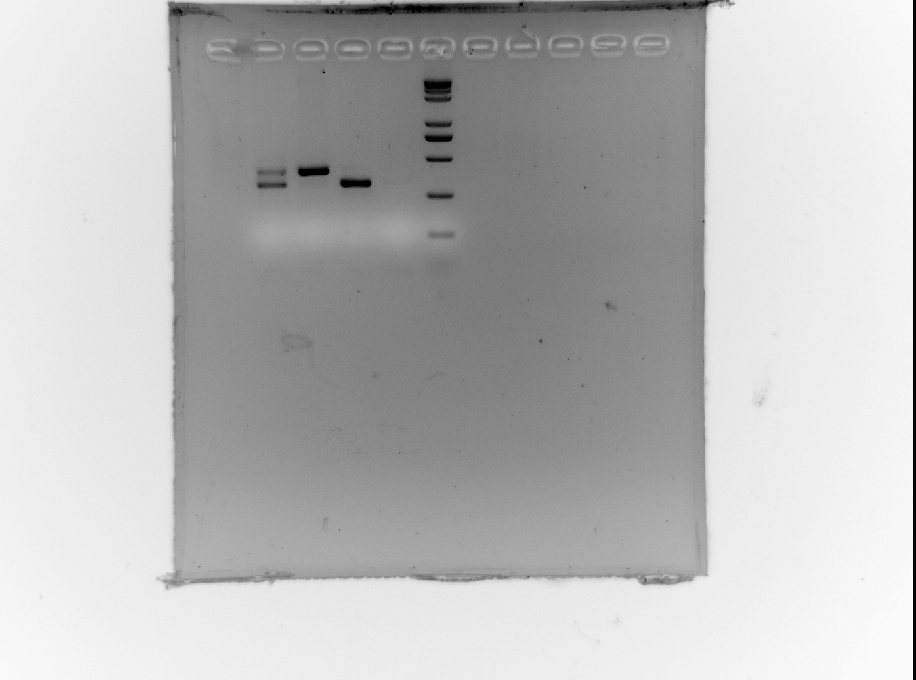

Supplement: Supplementary file 8 — Additional file 8. Images of the original, uncropped gels/blots. [file 12915_2023_1736_MOESM8_ESM.zip › Additional file 8.Images of the original, uncropped gelsblots/Fig 1C-flox.jpg]

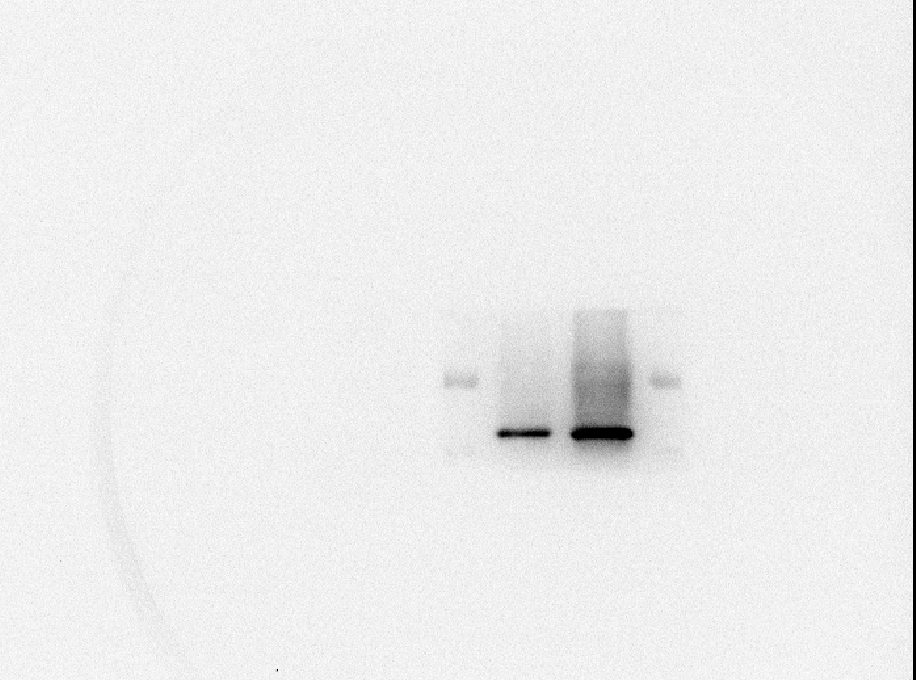

Supplement: Supplementary file 8 — Additional file 8. Images of the original, uncropped gels/blots. [file 12915_2023_1736_MOESM8_ESM.zip › Additional file 8.Images of the original, uncropped gelsblots/Fig 1E-ACTIN.jpg]

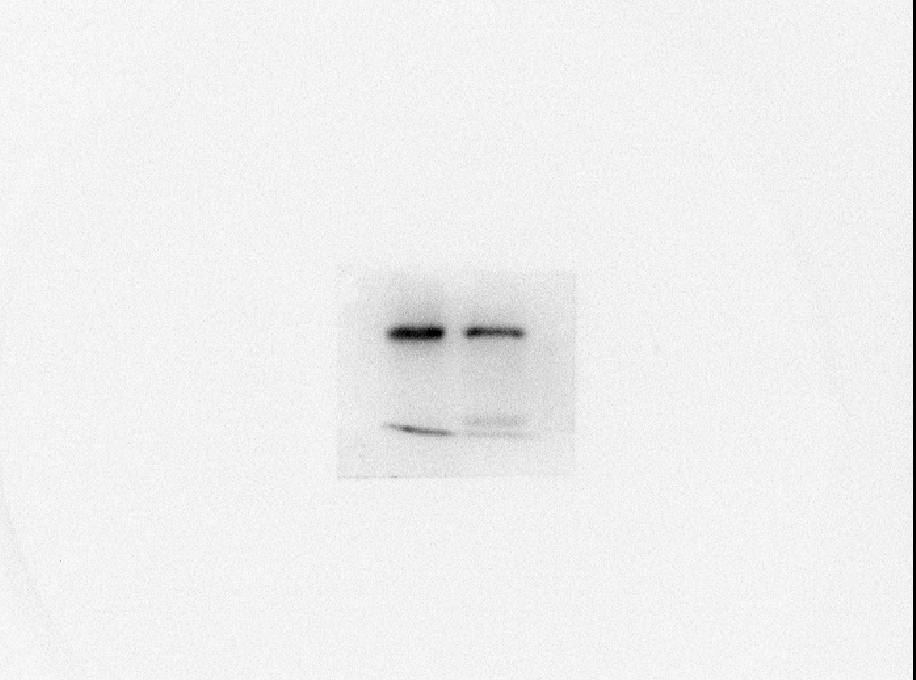

Supplement: Supplementary file 8 — Additional file 8. Images of the original, uncropped gels/blots. [file 12915_2023_1736_MOESM8_ESM.zip › Additional file 8.Images of the original, uncropped gelsblots/Fig 1E-SR2.jpg]

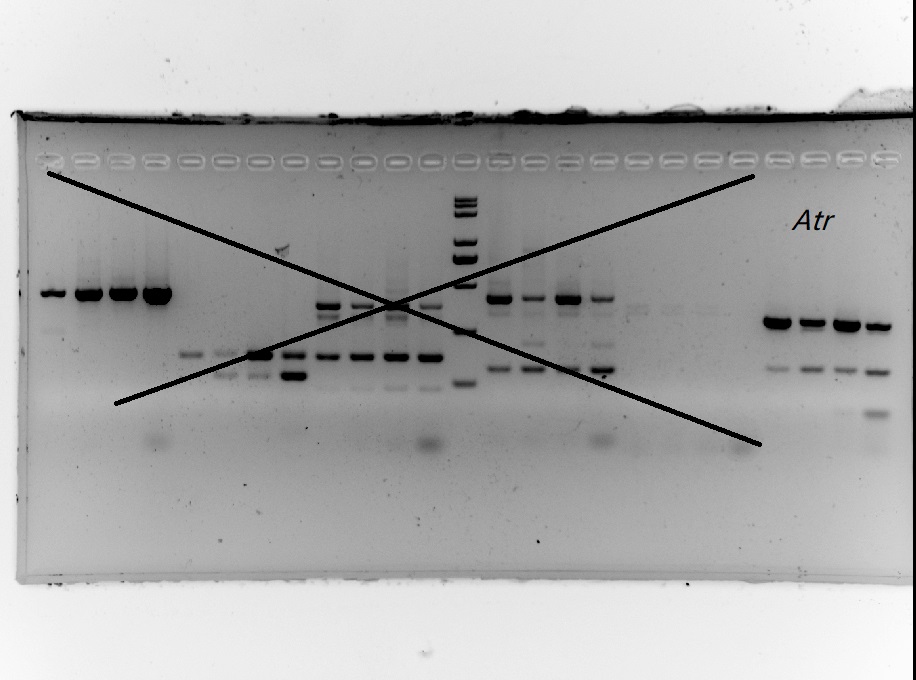

Supplement: Supplementary file 8 — Additional file 8. Images of the original, uncropped gels/blots. [file 12915_2023_1736_MOESM8_ESM.zip › Additional file 8.Images of the original, uncropped gelsblots/Fig 7E-Atr.jpg]
